# Editorial: Non-invasive Brain Stimulation for Neurodegenerative Disorders: From Investigation to Therapeutic Application

**DOI:** 10.3389/fneur.2022.820942

**Published:** 2022-03-03

**Authors:** Francesco Di Lorenzo, Antonio Oliviero, Andrea Guerra, Peter J. Fried

**Affiliations:** ^1^Department of Behavioral and Clinical Neurology, Santa Lucia Foundation Istituto di Ricerca e Cura a Carattere Scientifico, Rome, Italy; ^2^Functional Exploration and Neuromodulation of the Nervous System (FENNSI) Group, Hospital Nacional de Paraplejicos, Servicio de Salud de Castilla la Mancha (SESCAM), Toledo, Spain; ^3^Center for Clinical Neuroscience, Hospital “Los Madroños,” Brunete, Madrid; ^4^Istituto Ricovero e Cura a Carattere Scientifico (IRCCS) Neuromed, Pozzilli, Italy; ^5^Berenson-Allen Center for Noninvasive Brain Stimulation, Division of Interventional Cognitive Neurology, Beth Israel Deaconess Medical Center, Harvard Medical School, Boston, MA, United States

**Keywords:** neurodegenerative diseases, Alzheimer's disease, H-coil, transcranial direct current stimulation, repetitive transcranial magnetic stimulation, treatment, Parkinson's disease, transcranial Alternate Current Stimulation

The term “neurodegenerative disorders” encompasses different syndromes characterized by a progressive decline in nervous system functions and integrity. The most common symptoms of neurodegeneration vary from the decline of higher cortical functions, such as cognition and behavior, to sensorimotor system and gait abnormalities and autonomic dysfunction. As neurodegenerative disorders mainly affect older people, their burden is destined to dramatically increase due to the population aging at a fast rate worldwide. To date, the vast majority of neurodegenerative diseases are lacking adequate disease-modifying treatments, thus highlighting the urgent need for new and effective therapeutic strategies. In recent years, non-invasive brain stimulation (NIBS) techniques, such as transcranial magnetic stimulation (TMS) and transcranial electric current stimulation (TES), have been developed and are currently under investigation in patients with neurodegenerative disorders for both therapeutic and diagnostic purposes. Although preliminary evidence indicates promising effects of NIBS in neurodegenerative diseases, the therapeutic mechanisms of NIBS are not completely understood. Indeed, even if some data resemble the findings obtained experimentally on hippocampal slices of murine models (1, 2), there is still huge variability in terms of physiological, technical, and statistical factors accounting for intra- and inter-subject variability in healthy and pathological cohorts (3, 4). Moreover, the field of NIBS is in continuous development, providing new protocols of neuromodulation able to influence brain activity and behavior (5, 6).

We proposed a Research Topic aimed to gather more evidence about the role of NIBS as a tool to better understand the pathophysiological mechanisms leading to neurodegeneration (by investigating means of different cortical activity) and to discuss challenges and strategies for innovative therapeutic options based on neuromodulation.

Even in the absence of neurodegenerative disease pathologies, age-related changes in neurophysiology can result in subtle, yet measurable, declines in cognitive functions of older adults. In an exhaustive systematic review, Goldthorpe et al. aimed to clarify the current state of NIBS research in augmenting healthy older adults' memory. They analyzed the different approaches used, possible sites of stimulation, and the susceptibility of various memory domains to NIBS, resulting in a useful guide for researchers to better understand how to support older adults as their memory changes. Moreover, in a sham-controlled, randomized, double-blinded study, Brambilla et al. investigated the possible role of transcranial random noise stimulation (tRNS) applied bilaterally over the dorsolateral prefrontal cortices (DLPFC) for five sessions as add-on therapy to cognitive training in healthy elderly people. While tRNS did not have a universal effect on all domains of cognition across all subjects, there were specific improvements on certain measures depending on the age group.

Over the past few decades, many studies have used NIBS techniques to elucidate the pathophysiology of neurodegenerative disorders and improve diseases symptoms. In a review, Rawji et al. adopted a topographical approach to describe how TMS has been used for the investigation of neurodegenerative diseases that differ in their neuroanatomical axes, i.e., motor cortex—corticospinal tract (motor neuron diseases), non-motor cortical areas (dementias), and subcortical structures (Parkinsonisms). They also provide useful recommendations to better tailor future studies aimed at understanding neurodegenerative diseases' pathophysiology. Spagnolo et al. performed a double-blind, placebo-controlled, randomized study to evaluate the safety and efficacy of high-frequency deep rTMS with the bilateral H5-coil in patients with Parkinson's disease. Sixty patients were randomized in three treatment conditions: verum rTMS to both primary motor and pre-frontal cortices (M1-PFC), verum rTMS to M1 with sham rTMS to PFC, or sham rTMS to both M1 and PFC. Their promising data showed that M1-PFC and M1 rTMS with H-coil were safe and induced a significantly greater improvement compared to sham in UPDRS part III total score, tremor, and lateralized sub-scores in PD patients. In another double-blind, placebo-controlled, pilot study, Leocani et al. used the H2-coil to deliver deep rTMS in patients with probable Alzheimer's disease (AD). The researchers simultaneously stimulated the frontal-parietal-temporal lobes bilaterally. Their results suggest that this NIBS technique is feasible and safe in AD patients. In addition, rTMS with H2-coil might provide beneficial, even though transient, effects on ADAS-cog score after repeated stimulation sessions over time. In a novel approach, Bocci et al. conducted a sham-controlled pilot study to test the effects of anodal transcranial Direct Current Stimulation (tDCS) over the cerebellum in a group of patients with Huntington's disease (HD). The stimulation was applied for 5 days a week and the possible effects on motor scores were assessed using the Unified Huntington's Disease Rating Scale—part I (UHDRS-I). The results showed a significant improvement in the UHDRS-I score, particularly in the subitem “dystonia,” both at the end of the anodal tDCS treatment and 4 weeks later, suggesting a rationale for cerebellar neuromodulation in HD. Ranieri et al. provide an update on the state of the art of neuromodulation in Amyotrophic Lateral Sclerosis (ALS) and a critical appraisal of the rationale for the application/optimization of brain stimulation interventions, in the light of their interaction with ALS pathophysiological mechanisms. The authors concluded that, overall, the available studies suggest a possible efficacy of neuromodulation in determining a slight reduction of disease progression, related to the type, duration, and frequency of treatment, though current evidence remains preliminary. Finally, Bréchet et al. developed a protocol for patient-tailored home-based transcranial Alternate Current Stimulation (tACS) in Alzheimer's disease-related dementia (ADRD), with an instruction program to train a caregiver to deliver daily sessions of tACS that can be remotely monitored by the study team. In the context of this futuristic setting, the authors discuss the neurobiological rationale to modulate oscillations and a description of the study protocol also showing preliminary data obtained from two ADRD patients. These results demonstrate the feasibility of the approach and provide pilot evidence on the safety of the remotely monitored, caregiver-administered, home-based tACS intervention in ADRD.

Taken together, we believe that the findings obtained from these articles provide a comprehensive view on the use of NIBS tools in neurodegenerative disorders, encouraging the pursuit of better tailored investigative studies and large, adequately powered, and randomized controlled trials.

## Author Contributions

FD wrote this Editorial. AO, AG, and PF revised the final version of the article. All authors listed have made a substantial, direct, and intellectual contribution to the work and approved it for publication.

## Conflict of Interest

AO is co-founder of NEUREK SL, a spin-off company of the Fundación Hospital Nacional de Paraplejicos. The remaining authors declare that the research was conducted in the absence of any commercial or financial relationships that could be construed as a potential conflict of interest.

## Publisher's Note

All claims expressed in this article are solely those of the authors and do not necessarily represent those of their affiliated organizations, or those of the publisher, the editors and the reviewers. Any product that may be evaluated in this article, or claim that may be made by its manufacturer, is not guaranteed or endorsed by the publisher.
